# Integrative Multi-Omics and Network Pharmacology Analyses Reveal Potential Mechanisms of *Codonopsis pilosula* in Mountain Sickness

**DOI:** 10.3390/ijms27209256

**Published:** 2026-10-18

**Authors:** Yaqi Zhou, Jiamin Wu, Zhiwei Tan, Wanxin He, Wen Tan, Mingxue Fu, Baowei Bai, Hao Li

**Affiliations:** 1 State Key Laboratory Incubation Base for Conservation and Utilization of Bio-Resource in Tarim Basin, College of Life Sciences and Technology, Tarim University, Alar 843300, China; 2 College of Animal Science and Technology, Northwest A&F University, Yangling 712100, China; 3 College of Life Science and Technology, Beijing University of Chemical Technology, Beijing 100029, China; 4 College of Life Science and Technology, Xinjiang University, Urumqi 830000, China; 5 College of Biological Sciences, China Agricultural University, Beijing 100193, China

**Keywords:** *Codonopsis pilosula*, mountain sickness, network pharmacology, transcriptomics, machine learning, single-cell RNA sequencing, molecular docking

## Abstract

Mountain sickness comprises a spectrum of high-altitude disorders associated with hypobaric hypoxia, including acute mountain sickness (AMS) and high-altitude pulmonary edema (HAPE). Although *Codonopsis pilosula* has been widely used in traditional medicine for conditions related to hypoxia, its molecular basis in mountain sickness remains poorly understood. Here, we integrated network pharmacology, transcriptomic analysis, machine-learning-based feature selection, single-cell RNA sequencing, and molecular docking to explore potential molecular associations between *Codonopsis pilosula*-related candidate targets and mountain sickness. We identified a set of *Codonopsis pilosula*-associated candidate targets enriched in biological processes related to hypoxia response, oxidative stress, inflammation, and vascular regulation. Integrated transcriptomic analyses across high-altitude exposure, AMS, and HAPE revealed convergent alterations in immune-related and oxygen transport-associated programs. Machine-learning analysis further provided internal computational prioritization of candidate genes associated with high-altitude exposure, with recurrent selection across complementary algorithms within the analyzed paired cohort. Single-cell transcriptomic analysis provided additional cellular context for these candidates, revealing distinct expression patterns across peripheral blood and lung cell populations. Molecular docking further identified potential interactions between selected *Codonopsis pilosula* compounds and candidate proteins, with *MMP9*, *CA2*, and *BCL2* showing comparatively favorable predicted interactions. Together, these findings provide an exploratory framework for evaluating potential associations between *Codonopsis pilosula*-associated candidate targets and transcriptional and cellular responses to high-altitude hypoxia, and identify candidate molecular pathways and targets for further experimental investigation.

## 1. Introduction

High-altitude environments impose substantial physiological challenges primarily due to reduced atmospheric pressure and decreased oxygen availability. When human adaptation to hypobaric hypoxia is insufficient, a spectrum of altitude-related disorders may occur, including acute mountain sickness (AMS), high-altitude pulmonary edema (HAPE), and other severe altitude-related complications [1,2,3,4]. These disorders are characterized by complex pathological processes involving impaired oxygen homeostasis, oxidative stress imbalance, inflammatory activation, immune dysregulation, and vascular dysfunction [5,6,7]. Although considerable progress has been made in understanding the physiological adaptation mechanisms to hypoxia, the molecular networks underlying the development and progression of high-altitude-related diseases remain incompletely understood.

*Codonopsis pilosula* (Dangshen), a traditional medicinal herb, contains diverse bioactive components, including polysaccharides, saponins, sterols, polyacetylenes, and sesquiterpenoids [8]. It has been widely used for its antioxidative, anti-inflammatory, and immunomodulatory properties, which are mainly associated with its diverse bioactive constituents, particularly polysaccharides [9,10]. Recent studies have further demonstrated that specific compounds derived from *Codonopsis pilosula*, including polyacetylenes, sesquiterpenoids, and polysaccharides, exhibit anti-hypoxic activity and may regulate oxidative stress, mitochondrial metabolism, and immune responses under hypoxic conditions [9,11,12]. However, despite accumulating evidence supporting the biological activities of *Codonopsis pilosula*, its molecular mechanisms in high-altitude-related disorders remain largely unresolved. In particular, the common regulatory networks and key molecular nodes through which *Codonopsis pilosula* may influence different hypoxia-associated conditions have not been systematically characterized.

Mountain sickness represents a heterogeneous disease spectrum rather than a single pathological state. High-altitude exposure represents the environmental challenge, which may induce physiological acclimatization in healthy individuals, whereas AMS and HAPE represent distinct clinical manifestations associated with maladaptation to hypobaric hypoxia [1,2,3]. Although these conditions share common mechanisms, including hypoxia signaling, oxidative stress, immune alteration, and vascular injury, they may involve distinct molecular responses [5,6]. Therefore, investigations focusing on a single disease condition or a single biological pathway may not fully capture the multi-target and multi-pathway characteristics of *Codonopsis pilosula*. Furthermore, traditional network pharmacology approaches often rely on predicted compound–target interactions and database-derived information, which may provide limited insights into the cellular context and biological relevance of candidate targets [13,14]. In recent years, integrated multi-omics strategies have provided new opportunities for investigating the complex mechanisms of natural products in multifactorial diseases [15]. For example, network pharmacology combined with transcriptomic analysis has been applied to identify potential molecular targets and regulatory pathways of traditional medicines [16]. Integrated network pharmacology and multi-omics analyses have revealed potential mechanisms of herbal formulations in high-altitude-related diseases, demonstrating the value of systematic approaches for dissecting complex biological responses [17]. In addition, machine-learning approaches provide a complementary strategy for prioritizing robust candidate features from high-dimensional biological datasets [18]. Single-cell RNA sequencing provides additional resolution by revealing cell-type-specific expression patterns, allowing the investigation of potential mechanisms within distinct cellular populations [19,20].

In this study, we established an integrative framework combining network pharmacology, transcriptomic analysis, machine-learning-based feature selection, single-cell RNA sequencing, and molecular docking to explore potential molecular associations between *Codonopsis pilosula*-associated candidate targets and mountain sickness-related molecular features. Integrated multi-omics approaches have increasingly been applied to characterize complex biological processes and identify potential molecular regulators in multifactorial diseases [21]. Unlike previous studies focusing on individual compounds, isolated pathways, or single disease models, we systematically integrated transcriptomic alterations associated with high-altitude exposure, AMS, and HAPE to examine their overlap with *Codonopsis pilosula*-associated candidate targets across different hypoxia-related conditions. Furthermore, machine-learning approaches were applied to prioritize robust candidate genes, while single-cell transcriptomic analyses were performed to characterize the cellular distribution patterns of these targets and provide additional biological context. Molecular docking was subsequently used to evaluate the predicted interactions between selected *Codonopsis pilosula*-derived compounds and candidate target proteins. Collectively, this study provides a hypothesis-generating systems-level framework for prioritizing potential molecular associations between *Codonopsis pilosula*-related candidate targets and mountain sickness, and identifies candidates for future experimental validation.

## 2. Results

### 2.1. Candidate Compounds and Target Genes of Codonopsis pilosula

**Figure 1:**
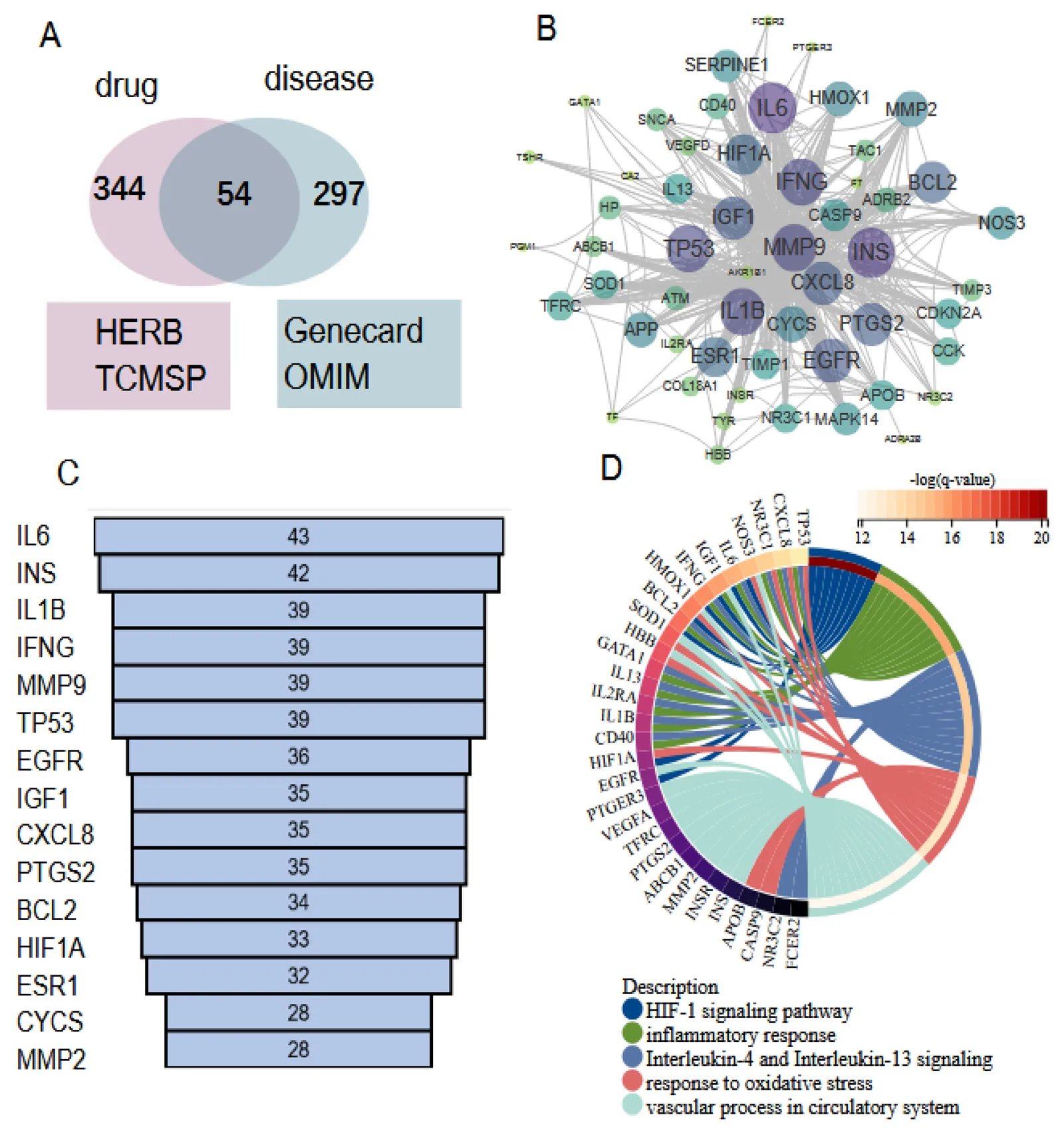
Identification and functional characterization of candidate targets shared between *Codonopsis pilosula* and mountain sickness. (**A**) Venn diagram showing the overlap between putative targets of *Codonopsis pilosula* and mountain sickness-related genes collected from GeneCards and OMIM databases. (**B**) Protein-protein interaction (PPI) network constructed using the intersecting genes in the STRING database. (**C**) Topological visualization of the PPI network in Cytoscape. Node size is proportional to the degree value. (**D**) Functional enrichment analysis of the intersecting genes. The top enriched biological pathways and processes are shown according to enrichment significance.

Based on the predefined oral bioavailability (OB) and drug-likeness (DL) thresholds, 21 candidate compounds were retained (Supplementary Table S1). After integrating target information from both databases, a total of 398 putative target genes were retained. In parallel, 351 mountain sickness-related genes were collected from the GeneCards and OMIM databases. Intersection analysis identified 54 overlapping candidate targets shared between *Codonopsis pilosula*-associated targets and mountain sickness-related genes (Supplementary Table S2, Figure 1A). Subsequently, a PPI network was constructed using the STRING database for these identified potential targets. The network data were imported into Cytoscape for network optimization and topological analysis (Figure 1B). Node degree was calculated to evaluate the relative importance of each target in the network, with node size representing the degree value (Figure 1C). Functional enrichment analysis demonstrated that these 54 targets are involved in multiple biological pathways and processes, including the HIF-1 signaling pathway, inflammatory responses, interleukin-4 and interleukin-13 signaling, response to oxidative stress, and vascular regulation in the circulatory system. These results indicate that the overlapping candidate targets are mainly associated with pathways related to hypoxia response, inflammation, oxidative stress, and vascular regulation (Figure 1D), providing a hypothesis-generating basis for further investigation of potential molecular associations between *Codonopsis pilosula* and mountain sickness. Notably, specific targets play critical roles in high-altitude diseases; for example, the mineralocorticoid receptor, encoded by the *NR3C2* gene, has been reported to play a critical role in the regulation of vascular tone in HAPE [22]. In addition, *MMP2* may contribute to lung structural remodeling under high-altitude hypoxic conditions [23]. These findings provided a rationale for further investigating potential molecular associations of *Codonopsis pilosula*-related candidate targets across three high-altitude-related conditions, including high-altitude exposure, AMS, and HAPE.

### 2.2. Codonopsis pilosula-Associated Target Genes in High-Altitude Exposure

**Figure 2:**
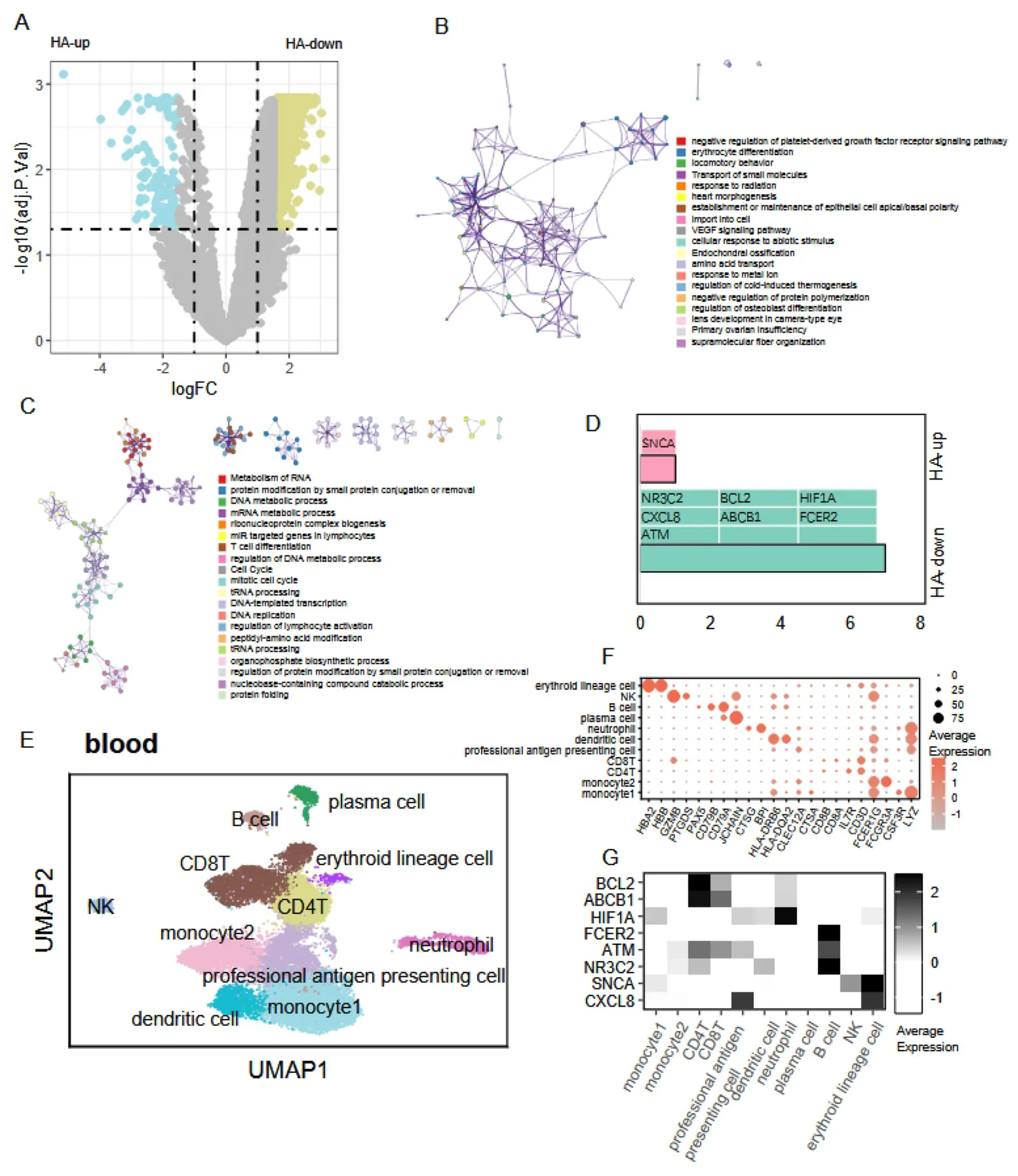
Transcriptomic characteristics of high-altitude exposure and candidate targets of *Codonopsis pilosula*. (**A**) Volcano plot showing DEGs between paired samples collected from 11 subjects before and after high-altitude exposure (22 samples). Differential expression was defined as adjusted *p* < 0.05 and |log2 fold change| > 1.5. (**B**) Functional enrichment analysis of upregulated genes. (**C**) Functional enrichment analysis of downregulated genes. (**D**) Bar charts showing the overlap between DEGs and predicted targets of *Codonopsis pilosula*. (**E**) UMAP visualization of peripheral blood single-cell RNA reference atlas data showing annotated cell populations. (**F**) Cell-type annotation of peripheral blood cell populations based on canonical marker genes. (**G**) Expression patterns of representative candidate target genes across annotated cell populations in the single-cell reference atlas.

Transcriptomic data were obtained from the publicly available GSE103940 dataset, which contains paired blood samples collected by the investigators of the original study before and after three days of high-altitude exposure. To explore the biological effects of high-altitude exposure, we first identified and analyzed transcripts exhibiting differential expression patterns between the two conditions. A total of 2782 differentially expressed genes (DEGs) were identified, including 118 upregulated and 2664 downregulated genes after high-altitude exposure compared with baseline/control samples, using the thresholds of adjusted *p* < 0.05 and |log2FC| > 1.5 (Figure 2A). Functional annotation demonstrated that the upregulated genes were predominantly associated with negative regulation of platelet-derived growth factor receptor signaling pathway (−log*p* = 4.795) and erythrocyte differentiation (−log *p*= 4.254) (Figure 2B). In contrast, the downregulated genes were significantly enriched in DNA and mRNA metabolic processes (−log *p* = 19.32/18.72), ribonucleoprotein complex biogenesis (−log *p* = 18.16), miRNA-targeted genes in lymphocytes (−log *p* = 14.59), and T cell differentiation (−log *p* = 14.10) (Figure 2C). These results suggest that high-altitude exposure is associated with erythroid-related changes and reduced expression of immune- and transcription-related gene programs.

By intersecting the 54 candidate genes with transcripts dysregulated by high-altitude exposure, we identified several key targets, including *SNCA* among the upregulated genes and *NR3C2*, *BCL2*, *HIF1A*, *CXCL8*, *ABCB1*, *FCER2*, and *ATM* among the downregulated genes (Figure 2D). These targets are functionally associated with immune regulation (*BCL2*, *CXCL8*), hypoxia response (*HIF1A*, *NR3C2*), and cell survival [24], suggesting that *Codonopsis pilosula* may be associated with molecular responses to high-altitude exposure.

We further characterized the cellular distribution patterns of these candidate targets using a human single-cell RNA reference atlas. The human single-cell reference atlas enabled the annotation of major peripheral blood cell populations, including CD8+ T cells, CD4+ T cells, and erythroid-lineage cells (Figure 2E,F). The reference atlas analysis showed that *SNCA* displayed preferential expression patterns in erythroid-lineage cells, whereas *BCL2* and *ABCB1* showed relatively higher expression patterns in CD4+ T-cell populations (Figure 2G). Previous studies have suggested that *SNCA* may participate in erythroid-related processes under stress conditions, including regulation of cellular homeostasis and adaptation responses [25]. In addition, *BCL2* is known to contribute to the maintenance of CD4+ T-cell survival [26]. The observed distribution of immune-related candidate genes in reference atlas cell populations, together with transcriptomic alterations after high-altitude exposure, suggests that immune regulation may represent an important component of high-altitude physiological and pathological responses. Taken together, these findings provide cellular context for candidate genes associated with erythroid adaptation and immune-cell survival, including *SNCA*, *BCL2*, and *ABCB1*.

### 2.3. Candidate Target Genes of Codonopsis pilosula Associated with High-Altitude Pulmonary Edema

**Figure 3:**
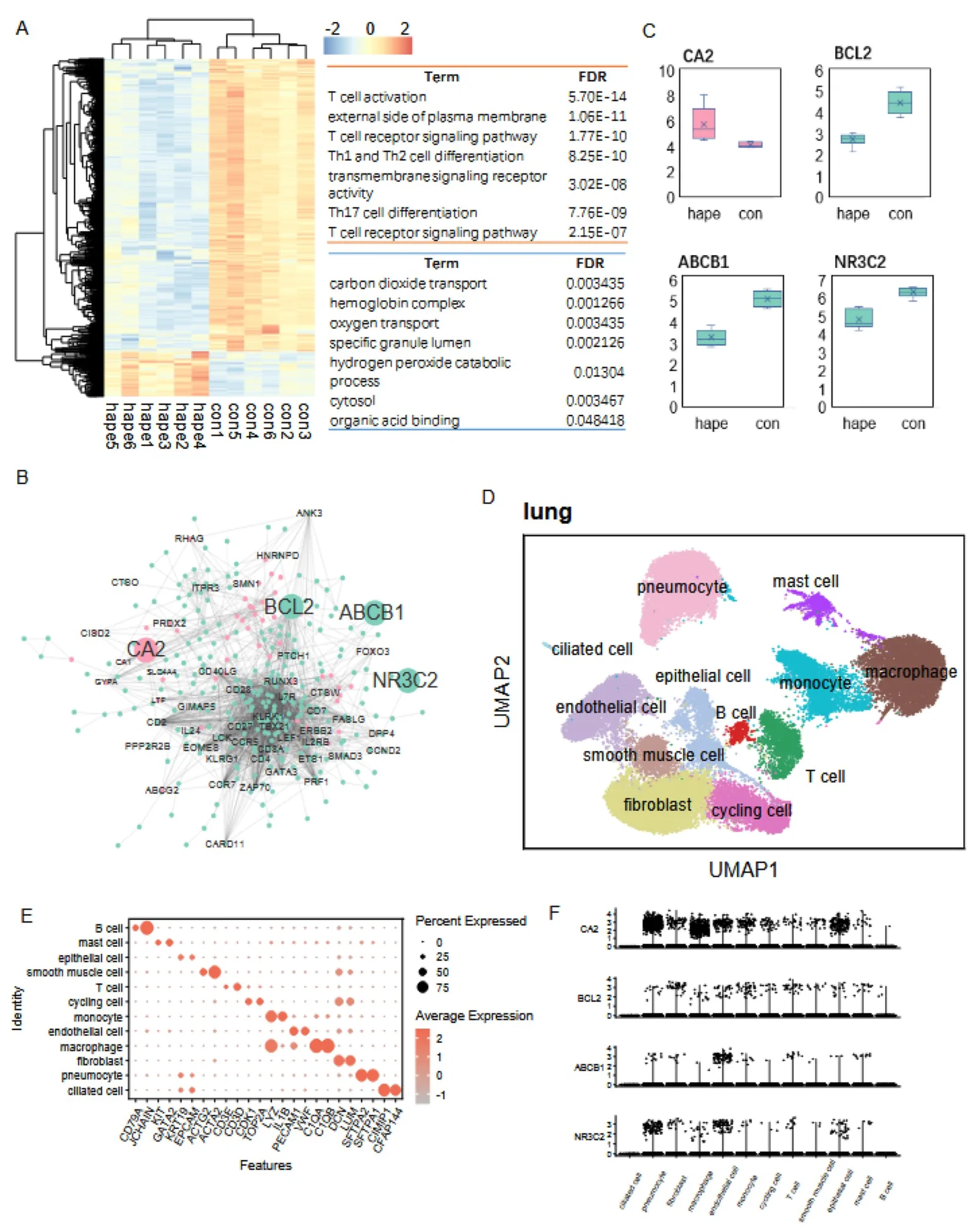
Transcriptomic characteristics and candidate targets of *Codonopsis pilosula* in HAPE. (**A**) Functional enrichment analysis of upregulated and downregulated genes identified in HAPE samples. The analysis included six HAPE samples and six control samples. (**B**) Protein-protein interaction network constructed from differentially expressed genes in HAPE. Pink indicates upregulated genes in the control group, whereas green indicates upregulated genes in the HAPE group. (**C**) Expression levels of selected target genes in HAPE and control samples. Differential expression was assessed using the limma (version 3.62.1) package with Benjamini–Hochberg correction. *p* values were 0.023 for *CA2*, 0.0006 for *BCL2*, 0.0004 for *ABCB1*, and 0.0003 for *NR3C2*. (**D**) UMAP visualization of lung cells from the human single-cell RNA reference atlas. (**E**) Cell-type annotation of lung cell populations based on canonical marker genes. (**F**) Expression patterns of *CA2* and *NR3C2* across annotated lung cell populations in the single-cell reference atlas.

Given that HAPE represents a severe form of high-altitude illness, we further investigated transcriptomic dysregulation in this pathological condition and explored candidate molecular associations between *Codonopsis pilosula*-related targets and HAPE-associated transcriptomic alterations. Comparative analysis revealed 477 DEGs between HAPE subjects and controls, including 63 upregulated and 414 downregulated genes. Functional enrichment analysis revealed distinct pathway alterations in HAPE. Downregulated genes were significantly enriched in T cell-related pathways, including T cell activation, T cell receptor signaling, and the differentiation of Th1, Th2, and Th17 cell subsets. This enrichment pattern was highly consistent with the observations in high-altitude exposure, suggesting that altered T cell-mediated immune responses may represent a common feature of high-altitude adaptation and disease progression [27]. In contrast, upregulated genes were predominantly associated with respiratory adaptation-related processes, including carbon dioxide transport, hemoglobin complex formation, and oxygen transport (Figure 3A). However, because bulk-blood transcriptomic signals may partly reflect differences in circulating cell composition (Supplementary Figure S1), these pathway-level changes cannot be attributed solely to transcriptional regulation within specific immune-cell populations.

To further explore the potential involvement of *Codonopsis pilosula* targets in HAPE-associated transcriptomic alterations, we constructed a PPI network based on the dysregulated genes. Network analysis showed that several putative *Codonopsis pilosula* target genes, including *CA2*, *BCL2*, *ABCB1*, and *NR3C2*, occupied central positions in the PPI network, and exhibited extensive interactions with immune-related genes, including *IL7R*, *IL2RB*, and *IL24* (Figure 3B). These centrally positioned candidate genes were also differentially expressed between HAPE subjects and controls including *CA2* (log2FC = 1.63, *p* value = 0.023), *BCL2* (log2FC = −1.50, *p* value = 0.0006), *ABCB1* (log2FC = −1.74, *p* value = 0.0004), and *NR3C2* (log2FC = −1.84, *p* value = 0.0003). Functionally, these genes are associated with hypoxia response and acid-base regulation (*NR3C2* and *CA2*) [28,29], apoptosis regulation (*BCL2*), barrier regulation and transporter activity (*ABCB1*), and immune modulation through their network connections with interleukin-related genes (Figure 3C).

We further characterized the cellular distribution of these candidate targets using lung cells from the human single-cell RNA reference atlas. UMAP visualization revealed major lung cell populations, including epithelial cells, endothelial cells, pneumocytes, macrophages, and other immune cells (Figure 3D,E). *CA2* exhibited a relatively broad expression pattern, with higher expression levels observed in pneumocytes, macrophages, and epithelial cells. In contrast, *NR3C2* showed detectable expression in pneumocyte and endothelial cell populations (Figure 3F). These reference atlas-based distribution patterns provide additional cellular context for interpreting the potential roles of *CA2* and *NR3C2* in hypoxia-associated pulmonary responses. *NR3C2*, which encodes the mineralocorticoid receptor (MR), may contribute to pulmonary vascular homeostasis by regulating endothelial inflammation, oxidative stress, vascular tone, and remodeling [22]. Together, these findings provide candidate molecular and cellular contexts linking *Codonopsis pilosula*-associated targets with gas exchange, immune regulation, vascular barrier integrity, and pulmonary epithelial-endothelial function in HAPE.

### 2.4. Machine-Learning Prioritization of Candidate Genes Associated with High-Altitude Exposure

Four complementary machine-learning algorithms were applied to the 39 expression-eligible genes from the predefined 54-gene candidate set. Model performance was evaluated using pooled outer leave-one-subject-out predictions, ensuring that both paired samples from each held-out subject were excluded from model fitting, hyperparameter tuning, and feature selection. LASSO achieved an ROC-AUC of 0.942 (95% CI, 0.835–1.000), Elastic Net 0.992 (95% CI, 0.967–1.000), Random Forest 0.983 (95% CI, 0.934–1.000), and SVM-RFE 1.000 (95% CI, 1.000–1.000). The corresponding balanced accuracy, sensitivity, and specificity were 0.909, 1.000, and 0.818 for LASSO, respectively, and 0.955, 1.000, and 0.909 for Elastic Net, Random Forest, and SVM-RFE, respectively (Supplementary Table S3). All four observed AUCs exceeded their paired-label permutation distributions (all *p* ≤ 0.004), indicating internal discrimination between baseline and high-altitude exposure samples within this paired cohort (Supplementary Figure S2 and Supplementary Table S3). These results represent internal subject-level validation only and should not be interpreted as evidence of predictive performance for AMS, HAPE, therapeutic response, or external populations.

**Figure 4:**
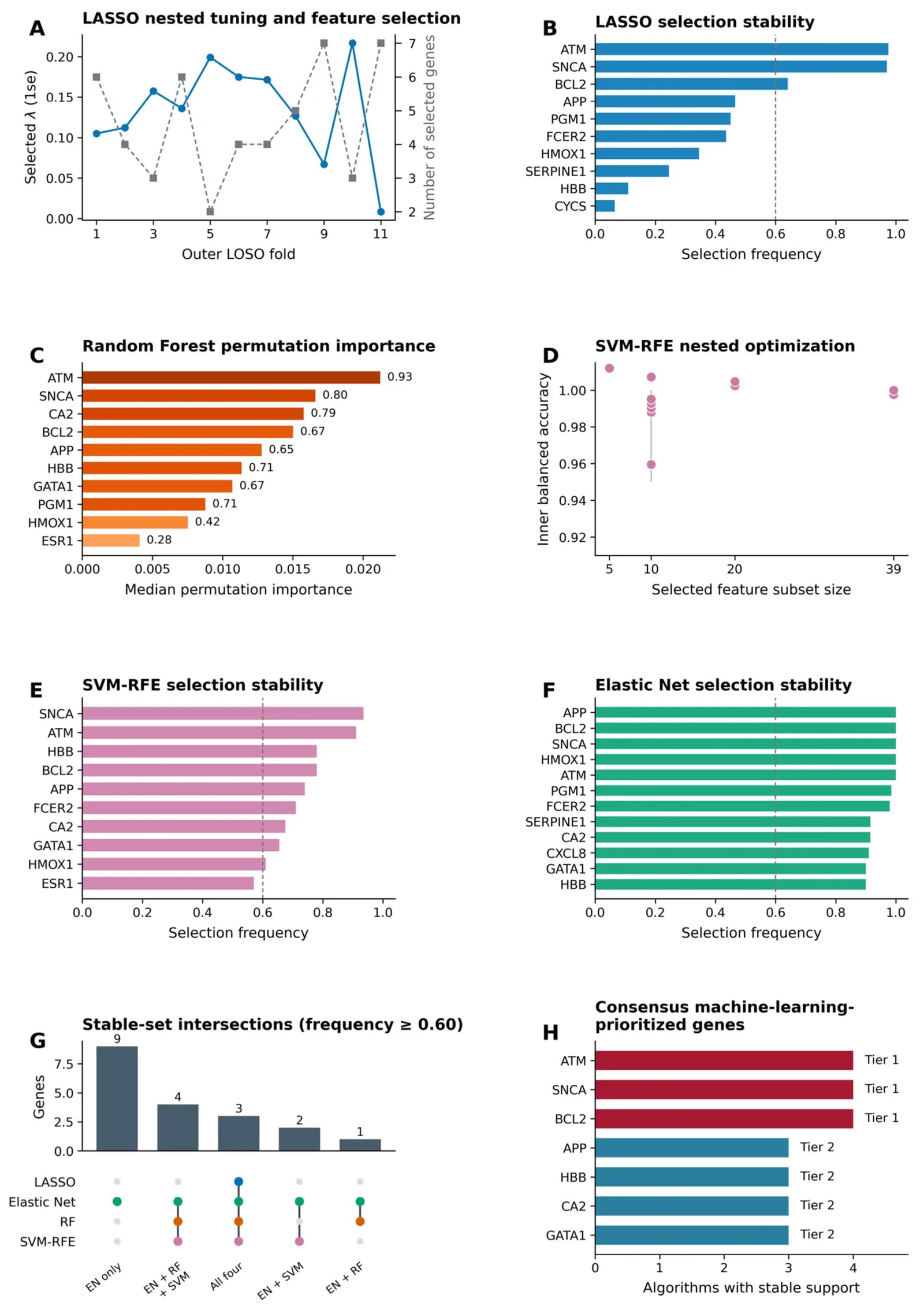
Machine-learning prioritization of candidate genes associated with high-altitude exposure. (**A**) LASSO nested tuning and feature selection across the 11 outer subject-level leave-one-subject-out folds. (**B**) LASSO selection frequency from 200 subject-level stability subsamples. (**C**) Random Forest raw out-of-bag permutation importance and permutation-based feature stability. (**D**) SVM-RFE nested optimization of the selected feature-set size. (**E**) SVM-RFE selection frequency from 200 subject-level stability subsamples. (**F**) Elastic Net selection frequency from 200 subject-level stability subsamples. (**G**) Intersections of stable feature sets across LASSO, Elastic Net, Random Forest, and SVM-RFE using the prespecified selection-frequency threshold of 0.60. Dot color indicates the machine-learning group. (**H**) Consensus prioritization according to the number of supporting algorithms. *ATM*, *SNCA*, and *BCL2* were supported by all four methods and classified as Tier 1 candidates, whereas *APP*, *HBB*, *CA2*, and *GATA1* were supported by three methods and classified as Tier 2 candidates. Model performance represents internal subject-level validation of baseline-versus-high-altitude-exposure classification using outer-fold held-out predictions and is reported in Supplementary Table S3.

Feature-selection behavior and stability across the four algorithms are summarized in Figure 4. LASSO showed fold-dependent variation in the selected feature set during nested tuning (Figure 4A), while repeated subject-level resampling identified *ATM*, *SNCA*, and *BCL2* as the most consistently selected LASSO features (Figure 4B). Random Forest permutation importance similarly prioritized *ATM*, *SNCA*, *CA2*, *BCL2*, *APP*, *HBB*, and *GATA1* (Figure 4C). SVM-RFE optimization favored compact feature subsets with high inner-validation accuracy (Figure 4D), and its stability analysis highlighted *SNCA*, *ATM*, *HBB*, *BCL2*, *APP*, *FCER2*, *CA2*, and *GATA1* among the repeatedly selected genes (Figure 4E). Elastic Net also yielded high selection frequencies for several overlapping candidates (Figure 4F).

Intersection analysis of the stable feature sets revealed recurrent support across algorithms (Figure 4G). *ATM*, *SNCA*, and *BCL2* met the prespecified stability criterion in all four methods and were classified as Tier 1 candidates, whereas *APP*, *HBB*, *CA2*, and *GATA1* were supported by three methods and were classified as Tier 2 candidates (Figure 4H). Therefore, recurrent selection across methods was interpreted as internal computational prioritization rather than independent biological validation.

### 2.5. Molecular Docking Analysis

**Figure 5:**
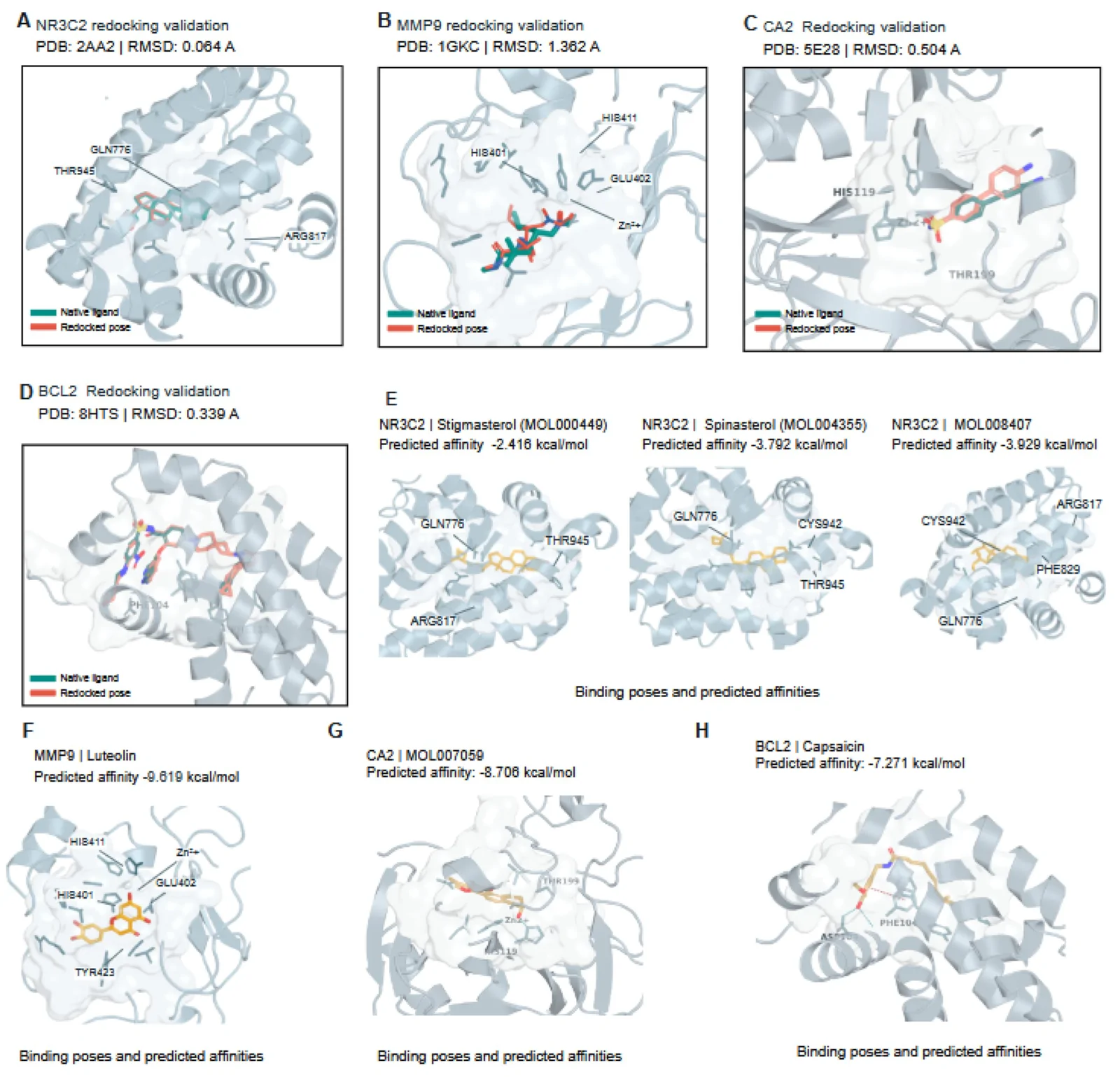
Molecular docking analysis of compounds with NR3C2, MMP9, CA2, and BCL2. (**A**–**D**) Redocking validation for NR3C2 (PDB ID: 2AA2), MMP9 (PDB ID: 1GKC), CA2 (PDB ID: 5E28), and BCL2 (PDB ID: 8HTS); the top-pose RMSD values are shown in the corresponding panels. Green indicates the native ligand and red the redocked pose; their overlap reflects the agreement between the docked and crystallographic binding modes. (**E**) Predicted binding poses and docking scores of stigmasterol (MOL000449), spinasterol (MOL004355), and stigmasterone (MOL008407) in the NR3C2 ligand-binding pocket. (**F**–**H**) Predicted binding poses and docking scores of luteolin (MOL000006) with MMP9, 3-beta-Hydroxymethyllenetanshiquinone (MOL007059) with CA2, and capsaicin (MOL002579) with BCL2. Docking scores are reported in kcal/mol to three decimal places.

Molecular docking was performed for NR3C2, MMP9, CA2, and BCL2 using validated receptor structures and redocking controls. The receptor structures were NR3C2 (PDB ID: 2AA2; chain A; resolution: 1.95 Å; pocket defined by aldosterone/AS4), MMP9 (PDB ID: 1GKC; chain A; resolution: 2.30 Å; pocket defined by NFH), CA2 (PDB ID: 5E28; chain A; resolution: 1.30 Å; pocket defined by BC5), and BCL2 (PDB ID: 8HTS; chain A; resolution: 1.25 Å; pocket defined by N2L). Redocking reproduced the reference ligand poses with top-pose RMSD values of 0.064, 1.362, 0.504, and 0.339 Å, respectively (Figure 5A–D). Under this docking protocol, stigmasterol (MOL000449), spinasterol (MOL004355), and stigmasterone (MOL008407) showed relatively weak predicted affinities for NR3C2 (−2.416, −3.792, and −3.929 kcal/mol, respectively). In contrast, luteolin (MOL000006) showed a more favorable predicted interaction with MMP9 (−9.619 kcal/mol), followed by 3-beta-Hydroxymethyllenetanshiquinone (MOL007059) with CA2 (−8.706 kcal/mol) and capsaicin with BCL2 (−7.271 kcal/mol) (Figure 5E–H).

## 3. Discussion

*Codonopsis pilosula* has been increasingly investigated for its anti-hypoxic, antioxidant, and immunomodulatory activities [30,31], but the molecular basis underlying these effects in high-altitude-related conditions remains incompletely characterized. In this study, we integrated network pharmacology with transcriptomic analysis across high-altitude exposure, AMS, and HAPE, and further combined machine-learning-based candidate prioritization, single-cell transcriptomic mapping, and molecular docking. This integrative strategy revealed a set of molecular processes and candidate genes associated with hypoxia, immune regulation, erythroid responses, and pulmonary function (Table 1), providing a broader view of the potential molecular links between *Codonopsis pilosula* and mountain sickness.

The network pharmacology analysis identified candidate *Codonopsis pilosula* targets that were enriched in processes related to hypoxia signaling, oxidative stress, inflammation, and vascular regulation. These processes represent interconnected components of the physiological response to reduced oxygen availability and are also involved in the development of altitude-related disorders [11,17]. Rather than being associated with a single signaling pathway, the candidate target network therefore reflects the multi-component and multi-process characteristics of *Codonopsis pilosula.* The transcriptomic analyses further connected this target landscape with molecular changes observed under different high-altitude conditions, suggesting that the potential effects of *Codonopsis pilosula* may involve several biological processes that are shared across hypoxic states. Because none of the transcriptomic datasets involved treatment with *Codonopsis pilosula* or its constituents, these overlaps should be interpreted as indirect, hypothesis-generating associations rather than evidence of treatment-induced transcriptional effects. A prominent feature of the transcriptomic analyses was the alteration of immune-related programs, particularly those associated with T-cell activation and differentiation, across high-altitude exposure and HAPE. At the same time, HAPE was characterized by increased representation of oxygen and carbon dioxide transport-related processes, consistent with the physiological demands imposed by hypobaric hypoxia. Together, these findings indicate that immune regulation and oxygen-handling processes are closely connected to the molecular response to high altitude. The marker-based analysis of bulk blood transcriptomes further suggested that changes in circulating cell composition may contribute to these patterns (Supplementary Text S1 and Supplementary Table S4). Thus, the observed immune-related changes likely reflect a combination of cellular composition and transcriptional responses [32], highlighting the importance of considering both aspects when interpreting blood-based transcriptomic alterations.

The integration of transcriptomic findings with candidate target information highlighted several genes with complementary molecular and cellular patterns. *NR3C2* was repeatedly identified across network and transcriptomic analyses and showed detectable expression in pneumocyte and endothelial cell populations in the lung reference atlas. As the gene encoding the mineralocorticoid receptor, *NR3C2* has been implicated in vascular homeostasis, inflammatory regulation, and oxidative stress responses [33]. Its distribution across pulmonary cell populations therefore provides a potential cellular context for its association with hypoxia-related pulmonary responses. In parallel, *CA2* and *BCL2* provided additional links to pulmonary and immune-related processes. *CA2* was associated with HAPE-related transcriptional changes and showed relatively broad expression across lung cell populations, whereas *BCL2* was recurrently prioritized by transcriptomic and machine-learning analyses and showed relatively higher expression in CD4+ T-cell populations. These patterns suggest that candidate genes identified in the present study may participate in distinct but interconnected aspects of high-altitude responses, including pulmonary homeostasis, immune regulation, and cellular adaptation.

The erythroid-related expression patterns of *HBB* and *SNCA* provide another perspective on the response to high altitude [34]. Both genes showed preferential expression in erythroid-lineage cells, consistent with the alteration of erythroid-related processes observed in the transcriptomic analyses. Increased demands on oxygen transport represent a central physiological challenge during high-altitude exposure, and erythroid adaptation is therefore an important component of the systemic response to hypoxia [24]. The cellular distribution of these genes provides additional context for linking the bulk transcriptomic findings to specific cell populations. In this framework, *SNCA* and *HBB* may represent molecular features associated with erythroid adaptation, whereas *BCL2* and *ABCB1* provide a complementary connection to immune-cell states.

Machine-learning analysis further refined the candidate landscape by identifying genes repeatedly selected across complementary algorithms. *ATM, SNCA,* and *BCL2* showed the most consistent cross-algorithm support, while *APP, HBB, CA2,* and *GATA1* were also recurrently selected. These findings indicate recurrent computational support for these candidates within the analyzed high-altitude exposure cohort. Importantly, the machine-learning analysis was based on a small paired cohort and lacked independent external validation [35]; therefore, the results should be interpreted as internal candidate prioritization rather than evidence of clinically generalizable biomarkers or prediction of AMS or HAPE. Together with the transcriptomic analyses, these results provide a structured basis for selecting candidate genes for subsequent biological investigation.

An exploratory co-expression analysis was also performed using the AMS dataset [36]. However, the network showed poor scale-free topology fit, and none of the module-AMS associations remained significant after multiple-testing correction. Given the small number of independent subjects and the repeated-measures design, the WGCNA results were considered descriptive only and were not used for candidate-gene prioritization or mechanistic inference. Detailed results are provided in the Supplementary Materials (Supplementary Figure S3, Method S1, Result S1). Separately, molecular docking showed a comparatively favorable predicted interaction between luteolin and *MMP9*, while *CA2* and *BCL2* also showed favorable predicted interactions with selected compounds. These structural predictions provide an additional compound-protein perspective and suggest candidate interactions for subsequent experimental investigation.

Taken together, the findings support a model in which the potential molecular associations of *Codonopsis pilosula* with mountain sickness are distributed across several interconnected biological processes rather than concentrated on a single target. Hypoxia response, oxygen transport, immune regulation, oxidative stress, and pulmonary vascular or epithelial functions emerged as recurring components of this molecular landscape. The combination of multi-condition transcriptomics, candidate-gene prioritization, and cell-type mapping further suggests that these processes operate across distinct cellular compartments, including erythroid, immune, epithelial, pneumocyte, and endothelial populations. This integrated view provides a framework for understanding how the diverse constituents of *Codonopsis pilosula* may interact with molecular responses associated with high-altitude exposure and its clinical manifestations.

Several aspects of the present study also define the scope of these findings. The network pharmacology and machine-learning analyses provide candidate prioritization based on available molecular and transcriptomic information, while the single-cell analysis provides cellular distribution rather than functional evidence. Similarly, bulk blood transcriptomes capture systemic responses but may integrate changes in both cell composition and cell-intrinsic transcription. The molecular docking results provide structural predictions of potential compound-protein compatibility, but do not establish biochemical binding, potency, efficacy, or downstream activity. These complementary approaches are therefore best considered together as a framework for prioritizing molecular relationships for future experimental studies. Functional experiments in relevant immune, erythroid, endothelial, and pulmonary cell models will help determine which of the identified candidate interactions contribute directly to the biological effects of *Codonopsis pilosula* under hypoxic conditions.

## 4. Materials and Methods

### 4.1. Identification of Codonopsis pilosula Constituents and Mountain Sickness-Related Targets

Candidate compounds were screened from locally archived TCMSP records [37] using oral bioavailability (OB) ≥ 30% and drug-likeness (DL) ≥ 0.18. Target information was integrated from the archived TCMSP and HERB records, followed by gene-symbol harmonization through basic text cleaning, exact-symbol matching, and duplicate removal [38]. After deduplication, 21 candidate compounds and 398 putative target genes were retained for subsequent analyses.

Mountain sickness-related genes were retrieved from GeneCards and Online Mendelian Inheritance in Man (OMIM) using the keyword “mountain sickness” [39,40]. The archived GeneCards export contained 307 unique gene symbols, while 55 unique gene symbols were retained from the OMIM records. Eleven genes were shared between the two databases, resulting in 351 unique disease-associated genes after merging and deduplication. Gene symbols were harmonized by basic text cleaning and exact-symbol matching before duplicate removal. The overlap between *Codonopsis pilosula*-associated targets and mountain sickness-related genes was considered a hypothesis-generating set of candidate associations for subsequent integrative analyses.

### 4.2. Protein–Protein Interaction Network Construction and Functional Enrichment Analysis

The overlapping genes between *Codonopsis pilosula*-associated targets and mountain sickness-related genes were submitted to the STRING database to construct a protein–protein interaction (PPI) network [41]. The resulting interaction network was imported into Cytoscape (version 3.9) [42] for visualization and topological analysis. Node degree was calculated to estimate the connectivity of individual genes within the network, with higher degree values indicating greater network connectivity.

The candidate targets were further subjected to functional enrichment analysis using Metascape. Enriched biological processes and signaling pathways were identified, and an adjusted *p* value < 0.01 was considered statistically significant [43].

### 4.3. Transcriptomic Datasets and Differential Gene Expression Analysis

Transcriptomic datasets were obtained from the Gene Expression Omnibus (GEO) database under accession numbers GSE103940 [44], GSE260910 [45] and GSE75665 [46]. These datasets were used to investigate gene-expression alterations associated with high-altitude exposure, acute mountain sickness (AMS), and high-altitude pulmonary edema (HAPE). None of these transcriptomic datasets involved treatment with *Codonopsis pilosula* or its constituent compounds; therefore, they were used to characterize disease- or exposure-associated transcriptional changes rather than direct treatment responses.

GSE103940 comprised 22 paired blood samples from 11 subjects collected before and after high-altitude exposure. GSE75665 comprised 20 repeated-measures samples from 10 subjects, including five AMS and five non-AMS individuals, with each subject sampled under plain and high-altitude conditions. GSE260910 comprised 12 independent samples, including six HAPE patients and six controls.

For GSE103940 and GSE75665, integer count matrices were analyzed using the limma-voom (version 3.62.1)workflow. Lowly expressed genes were filtered using filterByExpr function [47], followed by TMM normalization and voom transformation [48,49,50]. Paired or repeated-measures structures were incorporated into the corresponding design matrices according to the study design. For GSE260910, the author-provided processed expression matrix was used. According to the GEO record and the original study, sequencing reads were aligned to the hg38 reference genome and gene/transcript expression was quantified using StringTie, with DESeq2 used for the original differential-expression analysis. Differential expression statistics were adjusted using the Benjamini–Hochberg method, and genes with adjusted *p* < 0.05 and |log2FC| > 1.5 were considered differentially expressed. Heatmaps were generated using the pheatmap package (version 1.0.13), and volcano plots were generated using ggplot2 (version 4.0.2).

The identified differentially expressed genes (DEGs) were subsequently integrated with the predicted targets of *Codonopsis pilosula* to identify hypothesis-generating candidate associations between herb-related targets and molecular alterations observed in high-altitude-related conditions. To evaluate the potential contribution of blood-cell composition to bulk transcriptomic signals, representative lineage-associated marker genes for T cells, B cells, NK cells, neutrophils, monocytes, and erythroid/reticulocyte populations were examined across the available bulk datasets. Marker-score differences were tested within the corresponding study designs, and *p* values were adjusted using the Benjamini–Hochberg method. These marker scores were used only as proxies for potential compositional shifts and were not interpreted as quantitative estimates of cell fractions.

### 4.4. Single-Cell RNA Sequencing Data Analysis

The single-cell RNA-sequencing dataset GSE134355 was obtained from the GEO database [19]. This dataset was used as a human reference atlas to characterize the cellular distribution of candidate genes. The peripheral-blood reference dataset contained 17,331 cells from four adult donors, whereas the lung reference dataset contained 33,698 cells from five donors. Single-cell data processing and analysis were performed using the Seurat package (version 5.1.0) in R (version 4.4.0) [20]. Gene-expression matrices were imported into Seurat using the CreateSeuratObject function. Gene expression values were normalized using the LogNormalize method with a scale factor of 10,000. Highly variable genes were identified using the variance-stabilizing transformation (VST) method, and the top 2000 variable genes were selected for downstream analysis. Data were subsequently scaled using the ScaleData function, followed by principal component analysis (PCA). The first 50 principal components were used for dimensionality reduction and clustering analysis.

To reduce potential batch effects among samples, Harmony integration was performed based on the available sample information [51]. Cellular neighborhoods were identified using the FindNeighbors function, and cell clusters were determined using the FindClusters function with a resolution parameter of 0.5. Uniform Manifold Approximation and Projection (UMAP) was performed using Harmony-corrected dimensions for visualization of cellular populations.

Cluster-specific marker genes were identified using the FindAllMarkers function with parameters of min.pct = 0.25 and logfc.threshold = 0.25. Genes with *p* values < 0.05 were considered statistically significant markers. Cell-type annotation was performed based on canonical marker genes reported in the original dataset and previous literature. Peripheral blood and lung cell populations were annotated according to established lineage-specific markers.

To characterize the cellular distribution of candidate *Codonopsis pilosula*-associated targets, the expression patterns of prioritized genes were evaluated across annotated cell populations. Average expression levels and the proportion of cells expressing each candidate gene were calculated within each cell type and visualized using feature plots and dot plots.

### 4.5. Machine-Learning Analysis

Machine learning was performed using GSE103940 to prioritize predefined candidate genes associated with transcriptional responses to high-altitude exposure. The candidate set consisted of 54 *Codonopsis pilosula*-mountain-sickness-related targets defined independently by network pharmacology, of which 39 genes were retained after expression filtering. This upstream filtering and TMM/voom transformation were completed before machine-learning analysis and did not involve phenotype-dependent feature selection. The binary outcome was high-altitude exposure versus baseline. This classification task was designed to distinguish high-altitude exposure from baseline within the analyzed paired cohort and was not intended to predict AMS, HAPE, therapeutic response, or clinical outcomes. Internal predictive performance was evaluated using an outer 11-fold subject-level leave-one-subject-out (LOSO) design [52]. In each outer fold, both paired samples from one subject were withheld as the test set, while the remaining 10 subjects were used for model development. Within each outer-training set, zero-variance filtering, model-specific preprocessing, feature selection where applicable, and hyperparameter tuning were performed without access to the held-out subject. LASSO, Elastic Net, and SVM-RFE were tuned using grouped five-fold inner cross-validation, with paired samples from the same subject assigned to the same fold. Random Forest hyperparameters were selected using out-of-bag balanced accuracy within the outer-training data.

LASSO and Elastic Net were implemented using the glmnet package (version 5.0). LASSO used α = 1, whereas Elastic Net evaluated α values of 0.1, 0.3, 0.5, 0.7, and 0.9; both used 40 λ values with lambda.min.ratio = 0.01 and the one-standard-error rule for model selection. Random Forest was implemented using the randomForest package (version 4.7-1.2) with 500 trees and candidate mtry values of 1, 6, 13, and 19. Linear SVM-RFE was implemented using the e1071 package (version 1.7-17) and evaluated cost values of 0.01, 0.1, 1, and 10 together with feature-set sizes of 2, 5, 10, 20, and 39 [53,54,55]. Feature ranking for SVM-RFE was recalculated within each inner-training fold to avoid information leakage.

Feature-selection stability was further assessed using 200 subject-level subsamples, each containing 9 of the 11 subjects while retaining paired samples. For LASSO, Elastic Net, and SVM-RFE, selection frequency was defined as the proportion of subsamples in which a gene was selected. Random Forest importance was quantified using raw out-of-bag permutation importance, corresponding to unscaled mean decrease in accuracy. A gene was considered supported by Random Forest only when its observed importance was positive and exceeded its gene-specific 95th percentile derived from structure-preserving label permutations. A prespecified selection-frequency threshold of 0.60 was used to define stable features. Genes supported by all four algorithms were classified as Tier 1 candidates, whereas those supported by three algorithms were classified as Tier 2 candidates.

Outer-fold predictions were pooled only after each subject had served once as the held-out test subject. ROC-AUC, balanced accuracy, sensitivity, and specificity were calculated from these out-of-fold predictions. Statistical significance was assessed using 499 paired-label permutation tests in which baseline/high-altitude labels were swapped within subjects and the full nested LOSO procedure was repeated. AUC uncertainty was estimated using 5000 subject-level bootstrap resamples of the frozen out-of-fold predictions while retaining paired samples. These bootstrap confidence intervals therefore quantify uncertainty in the observed out-of-fold predictions rather than variability from repeated model retraining.

### 4.6. Molecular Docking

Molecular docking was performed to evaluate potential interactions between selected candidate compounds and four prioritized targets: NR3C2, MMP9, CA2, and BCL2. The docked compound-target pairs were selected from the upstream network-pharmacology results to represent candidate associations involving targets supported by the integrative analyses or high-altitude disease relevance, while also requiring the availability of suitable co-crystallized receptor structures for structure-based docking. Receptor structures were obtained from the Protein Data Bank [56] as follows: NR3C2 (PDB ID: 2AA2; chain A; resolution: 1.95 Å), MMP9 (PDB ID: 1GKC; chain A; resolution: 2.30 Å), CA2 (PDB ID: 5E28; chain A; resolution: 1.30 Å), and BCL2 (PDB ID: 8HTS; chain A; resolution: 1.25 Å). Docking pockets were defined according to the corresponding co-crystallized ligands identified by their PDB chemical component IDs: AS4 (aldosterone) for NR3C2, NFH for MMP9, BC5 (4′-aminobiphenyl-4-sulfonamide) for CA2, and N2L (the compound S-10r) for BCL2 [56]. The catalytic Zn^2+^ and Ca^2+^ ions in MMP9 and the catalytic Zn^2+^ ion in CA2 were retained during receptor preparation; no metal ions were present in the BCL2 structure.

Three-dimensional ligand structures were obtained from PubChem using the following compound records: stigmasterol (PubChem CID: 5280794), spinasterol (PubChem CID: 5281331), stigmasterone (PubChem CID: 14807783), luteolin (PubChem CID: 5280445), 3-beta-Hydroxymethyllenetanshiquinone (PubChem CID: 5318290), and capsaicin (PubChem CID: 1548943), and were subjected to MMFF94 energy minimization before docking. Molecular docking was performed using AutoDock Vina version 1.2.7 [57]. The docking grid centers and box sizes were as follows: NR3C2, center (18.823, 72.533, 20.402), box size 22 Å × 22 Å × 22 Å; MMP9, center (65.839, 30.707, 117.746), box size 22 Å × 22 Å × 22 Å; CA2, center (−3.770, 4.124, 14.906), box size 22 Å × 22 Å × 22 Å; and BCL2, center (−0.210, 9.374, 7.186), box size 26 Å × 27 Å × 27 Å. Candidate-compound docking was performed with an exhaustiveness of 32. For CA2 and BCL2, 20 binding modes were retained, with an energy range of 8 kcal/mol and a random seed of 20260917; redocking was performed with an exhaustiveness of 64.

To assess docking reproducibility, the native co-crystallized ligands were redocked into their respective binding pockets. The top-pose RMSD values were 0.064 Å for NR3C2, 1.362 Å for MMP9, 0.504 Å for CA2, and 0.339 Å for BCL2, all below the commonly used 2.0 Å acceptance threshold. The top-ranked poses were used for subsequent interaction analysis and visualization [58].

## 5. Conclusions

This study provides a systems-level, hypothesis-generating framework for examining potential molecular associations between *Codonopsis pilosula* and mountain sickness. By integrating network pharmacology with transcriptomic analyses across high-altitude exposure, AMS, and HAPE, we prioritized candidate genes and biological processes related to hypoxia response, immune regulation, erythroid adaptation, oxidative stress, and pulmonary function. Machine-learning analysis further prioritized recurrent candidates, single-cell transcriptomic mapping provided cellular context, and molecular docking generated predicted compound-protein interactions. These findings should be interpreted as computationally generated hypotheses rather than evidence of therapeutic efficacy or experimentally established mechanisms. Further biochemical binding assays, relevant cell models, perturbation studies, and in vivo validation will be required to determine whether the prioritized compounds and candidate genes have functional roles in high-altitude hypoxia or mountain sickness.

## Figures and Tables

**Table 1:** Summary of major findings and potential mechanisms of *Codonopsis pilosula* in mountain sickness.

Condition/Analysis	Main Findings	Key Candidate Genes	Potential Biological Implication
High-altitude exposure	Downregulation of T-cell-related pathways; alteration of erythroid-related processes	*SNCA*, *BCL2*, *ABCB1*, *HIF1A*, *NR3C2*	Erythroid adaptation and immune regulation
HAPE	Downregulation of T-cell-related pathways and upregulation of oxygen/CO_2_ transport-related processes	*CA2*, *BCL2*, *ABCB1*, *NR3C2*	Gas exchange, vascular regulation, and immune modulation
Machine learning	Recurrent candidate selection across four algorithms	Tier 1: *ATM*, *SNCA*, *BCL2;* Tier 2: *APP*, *HBB*, *CA2*, *GATA1*	Consistent cross-algorithm candidate prioritization within the analyzed cohort
Molecular docking	Favorable predicted interactions were observed for luteolin–*MMP9*, 3-beta-Hydroxymethyllenetanshiquinone (MOL007059)–CA2, and capsaicin–*BCL2*; weaker predicted interactions were observed for the tested NR3C2 compounds	*MMP9*, *CA2*, *BCL2*, *NR3C2*	Predicted compound–protein compatibility requiring experimental validation

## Data Availability

The datasets analyzed in this study are publicly available in the Gene Expression Omnibus (GEO) repository under accession numbers GSE103940, GSE260910, GSE75665, and GSE134355. Analysis scripts, processed intermediate files, documented random seeds, model and validation outputs, molecular-docking configuration files and outputs, and compound/target provenance records associated with this study are publicly available through the GitHub reproducibility repository (version V1.0.3) (https://github.com/leehao-create/codonopsis-mountain-sickness-reproducibility; accessed on 17 October 2026). A frozen version of the final reproducibility package has been permanently archived in Zenodo (DOI: 10.5281/zenodo.23343343). The archived Zenodo release is version V1.0.3.

## Supplementary Materials

The following supporting information can be downloaded at: https://www.mdpi.com/article/10.3390/ijms27209256/s1.
