# Supplementary material for: Integrative Multi-Omics and Network Pharmacology Analyses Reveal Potential Mechanisms of Codonopsis pilosula in Mountain Sickness
Source: Int J Mol Sci. 2026 Oct 18;27(20):9256. doi: 10.3390/ijms27209256 (PMC13705243; doi:10.3390/ijms27209256)
Supplement: Supplementary file 1 [file ijms-27-09256-s001.zip › Supplementary Text.pdf]

**Supplementary Text S1. Marker-based analyses of the bulk transcriptomic datasets**

To assess whether the observed immune-related transcriptional patterns might be influenced by changes in blood-cell composition, we examined representative lineage-associated marker genes in the bulk transcriptomic datasets. In GSE103940, the T-cell marker score was significantly decreased after high-altitude exposure (effect = −0.936, BH-adjusted P = $5.82 \times 10^{-4}$), whereas the erythroid/reticulocyte marker score was significantly increased (effect = 1.420, BH-adjusted P = $1.23 \times 10^{-4}$). Consistent decreases were observed for T-cell-associated genes including CD3D, CD3E, CD3G, and IL7R, while erythroid-associated markers showed a coordinated increase. In GSE75665, none of the tested lineage-marker scores remained significant after multiple-testing correction. Because these marker-based analyses do not provide direct estimates of cell proportions, the results were interpreted as evidence that cellular-composition changes may contribute to some of the bulk transcriptomic signals rather than as direct measurements of cell abundance (Supplementary Tables S4A–S4E and Supplementary Figure S1).

**Supplementary Methods S1. Exploratory Weighted Gene Co-Expression Network Analysis**

WGCNA was performed using the WGCNA package in R to explore gene co-expression patterns and module-AMS relationships [36]. After genes with missing values or zero expression were removed, 20,751 genes remained. Genes with MAD ≤ 1.5 were excluded, leaving 2,810 genes. All retained samples and genes passed the goodSamplesGenes check. Sample outliers were assessed using average-linkage hierarchical clustering; no formal cutoff was applied, and no sample was excluded. Soft-threshold selection was performed under a signed-network setting, and module construction was carried out using blockwiseModules with power = 16, TOM type = signed, minimum module size = 20, merge cut height = 0.05, reassign threshold = 0, and maximum block size = 2,810. At $\beta = 16$, the scale-free topology fit index was $R^2 = 0.0419$, which did not reach the conventional threshold of 0.85. Because none of the evaluated powers achieved a satisfactory scale-free topology fit, $\beta = 16$ was retained solely to construct an exploratory descriptive network; the resulting network was not interpreted as satisfying the scale-free topology criterion. Genes were hierarchically clustered using TOM dissimilarity, and modules were identified by dynamic tree cutting. Module eigengenes represented the overall expression patterns of individual modules. Pearson correlations between module eigengenes and AMS status (AMS = 1, non-AMS = 0) were calculated, and P values were adjusted using the Benjamini-Hochberg method. For highlighted genes, intramodular connectivity (kWithin) was calculated from the signed weighted adjacency matrix as the sum of connection strengths with other genes in the same module. Signed module membership (kME) and within-module rank and percentile were also calculated to account for differences in module size.

Repeated measurements from the same subjects were retained as individual observations in the exploratory network construction, and within-subject dependence

was not explicitly modeled in the WGCNA framework.Given the small cohort size, the poor scale-free topology fit ($R^2 = 0.0419$), and the repeated-measures structure of the dataset, this analysis was treated strictly as exploratory and descriptive and was not used for candidate-gene prioritization or mechanistic inference.

**Supplementary Results S1. Exploratory Co-Expression Analysis in the AMS Dataset**

To investigate the co-expression context of *Codonopsis pilosula*-associated candidate genes in AMS, WGCNA was performed to explore co-expression modules potentially related to AMS status. Hierarchical clustering based on a topological overlap matrix (TOM) dissimilarity measure (1-TOM) revealed 12 distinct gene modules, each representing a set of co-expressed genes with potential functional synergy (Supplementary Figure S3A and B).

Module–trait correlation analysis was subsequently performed using a binary AMS phenotype (AMS = 1, non-AMS = 0) to evaluate the association between co-expression modules and AMS status (Supplementary Figure S3C). Several modules showed moderate positive correlations with AMS status, including MEmagenta ($r = 0.56$, $P = 0.010$), MEgreenyellow ($r = 0.59$, $P = 0.006$), MEblack ($r = 0.53$, $P = 0.016$), MEbrown ($r = 0.50$, $P = 0.025$), and MEyellow ($r = 0.50$, $P = 0.026$). However, none of these associations remained significant after Benjamini–Hochberg correction. Therefore, we interpreted the WGCNA results as exploratory evidence of trends in AMS-related co-expression patterns, rather than definitive evidence for disease-associated modules.

To further investigate the relationship between *Codonopsis pilosula*-associated targets and AMS-related co-expression networks, candidate target genes were mapped to WGCNA modules. Among the 54 putative target genes, 13 genes were assigned to WGCNA modules, including *HMOX1, HIF1A, HBB, MAPK14, IL1B,* and *MMP9* in the turquoise module, *AKR1B1, TP53, FCER2,* and *TIMP1* in the blue module, *GATA1* in the brown module, SNCA in the green module, and SOD1 in the red module (Supplementary Figure S3D). These results provide additional information regarding the co-expression context of candidate targets and their potential involvement in AMS-related transcriptional regulation. Among these genes, *MAPK14, HIF1A,* and *TP53* had the highest intramodular connectivity. MMP9 showed moderate-to-low intramodular connectivity (kWithin = 226.2; rank 967/1,147) but a high signed kME (0.852), indicating that it was associated with the turquoise module eigengene without acting as a high-connectivity hub (Supplementary Figure S3E).

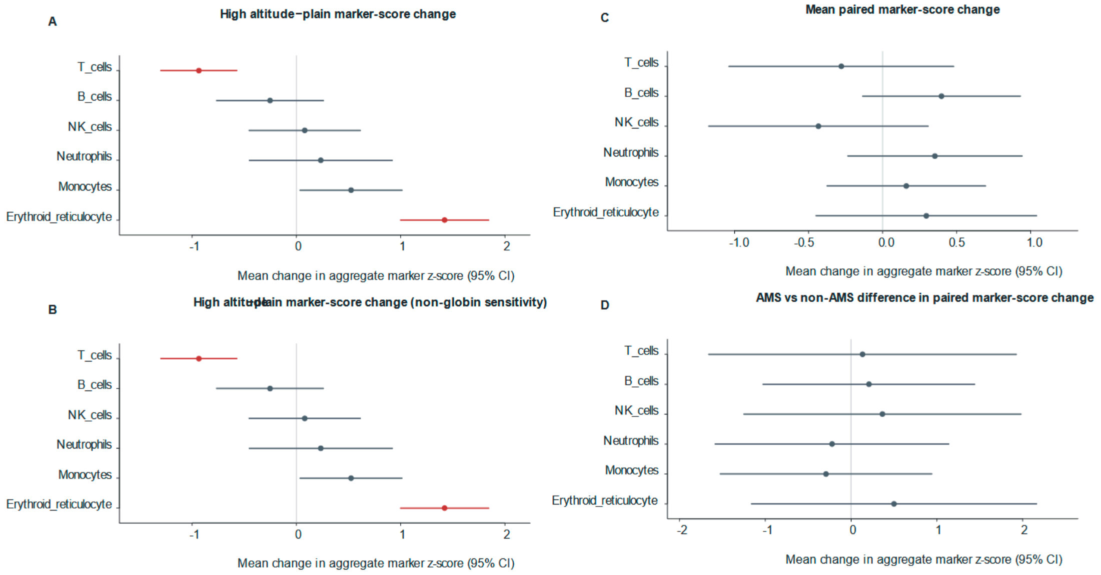


**Figure S1.** Marker-based cell-composition audit of bulk blood transcriptomic datasets
(A) High altitude − plain marker-score change.
(B) High altitude− plain marker-score change (non-globin sensitivity).
(C) Mean paired marker-score change.
(D) AMS vs non-AMS difference in paired marker-score change.

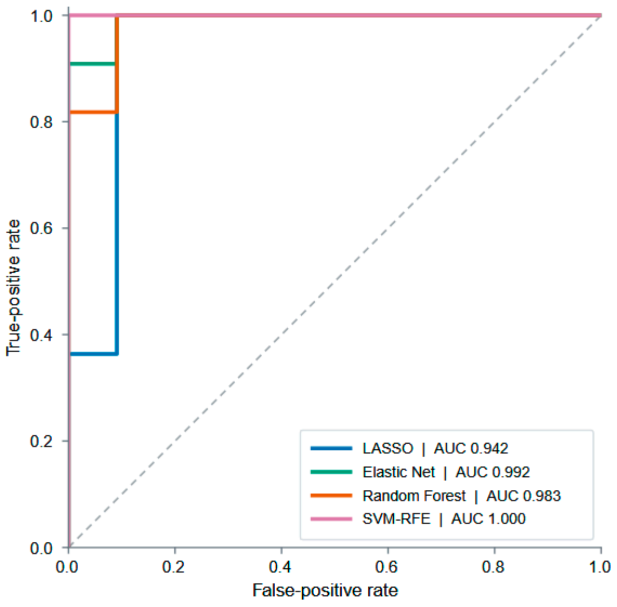


**Figure S2.** Outer leave-one-subject-out out-of-fold ROC performance of the four machine-learning models.

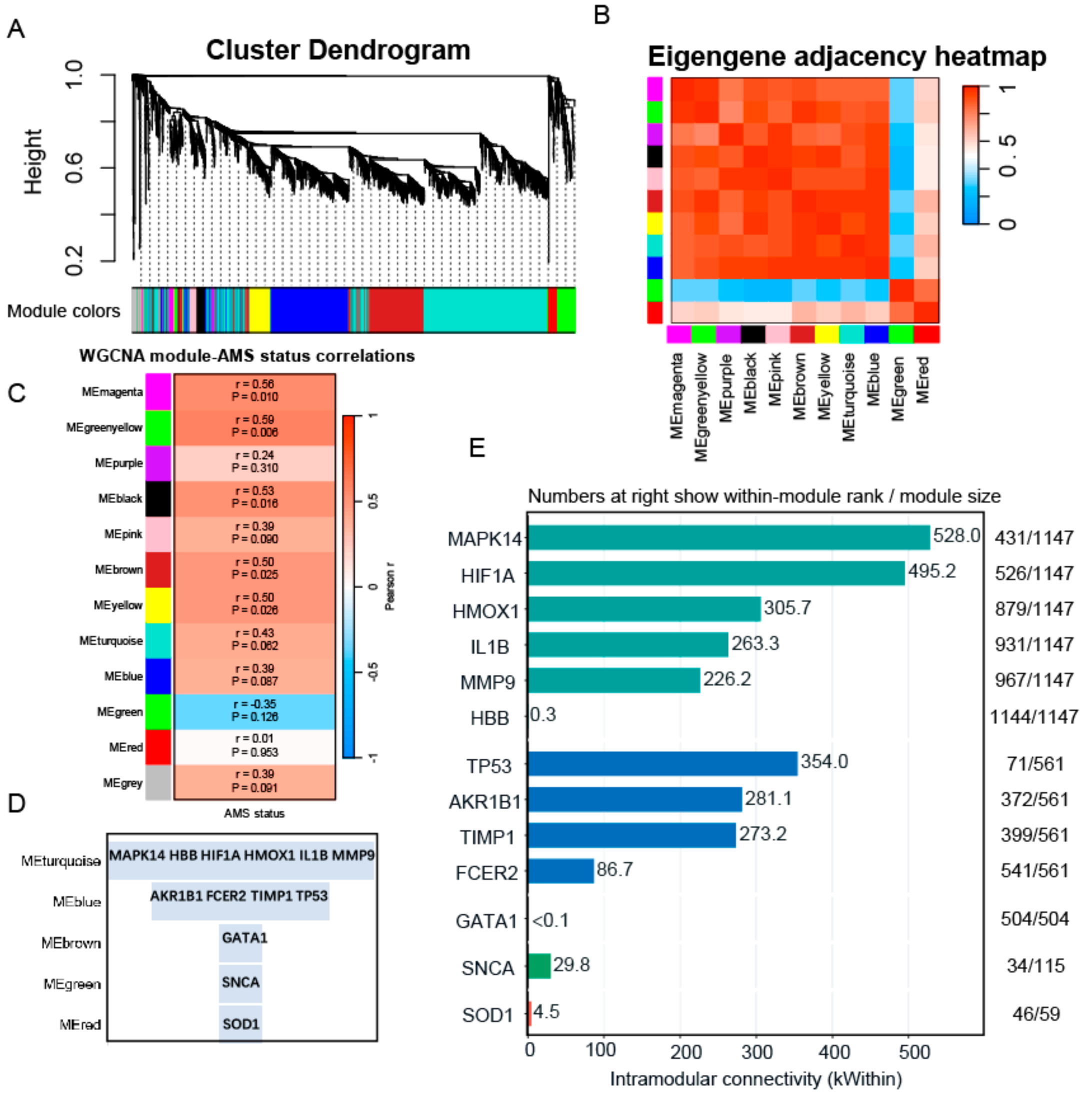


**Figure S3.** WGCNA identifies co-expression patterns associated with acute mountain sickness (AMS).

(A) Gene dendrogram and module assignment based on topological overlap matrix (TOM) dissimilarity. The color bar indicates the module assignment of individual genes.

(B) Eigengene adjacency heatmap showing the pairwise relationships among WGCNA module eigengenes.

(C) Module–trait correlation analysis between WGCNA modules and binary AMS status (AMS = 1, non-AMS = 0). Pearson correlation coefficients (r) and corresponding nominal P values are shown. None of the modules remained significant after Benjamini–Hochberg correction

(D) Distribution of *Codonopsis pilosula*-associated candidate target genes across WGCNA modules.

(E) Intramodular connectivity (kWithin; sum of signed weighted adjacency values with other genes in the same module) of highlighted genes. Colors indicate WGCNA modules, and numbers at right show within-module rank/module size.
