# Supplementary material for: Integrative Multi-Omics and Network Pharmacology Analyses Reveal Potential Mechanisms of Codonopsis pilosula in Mountain Sickness
Source: Int J Mol Sci. 2026 Oct 18;27(20):9256. doi: 10.3390/ijms27209256 (PMC13705243; doi:10.3390/ijms27209256)
Supplement: Supplementary file 1 [file ijms-27-09256-s001.zip › Supplementary_Figure_S1.pdf]

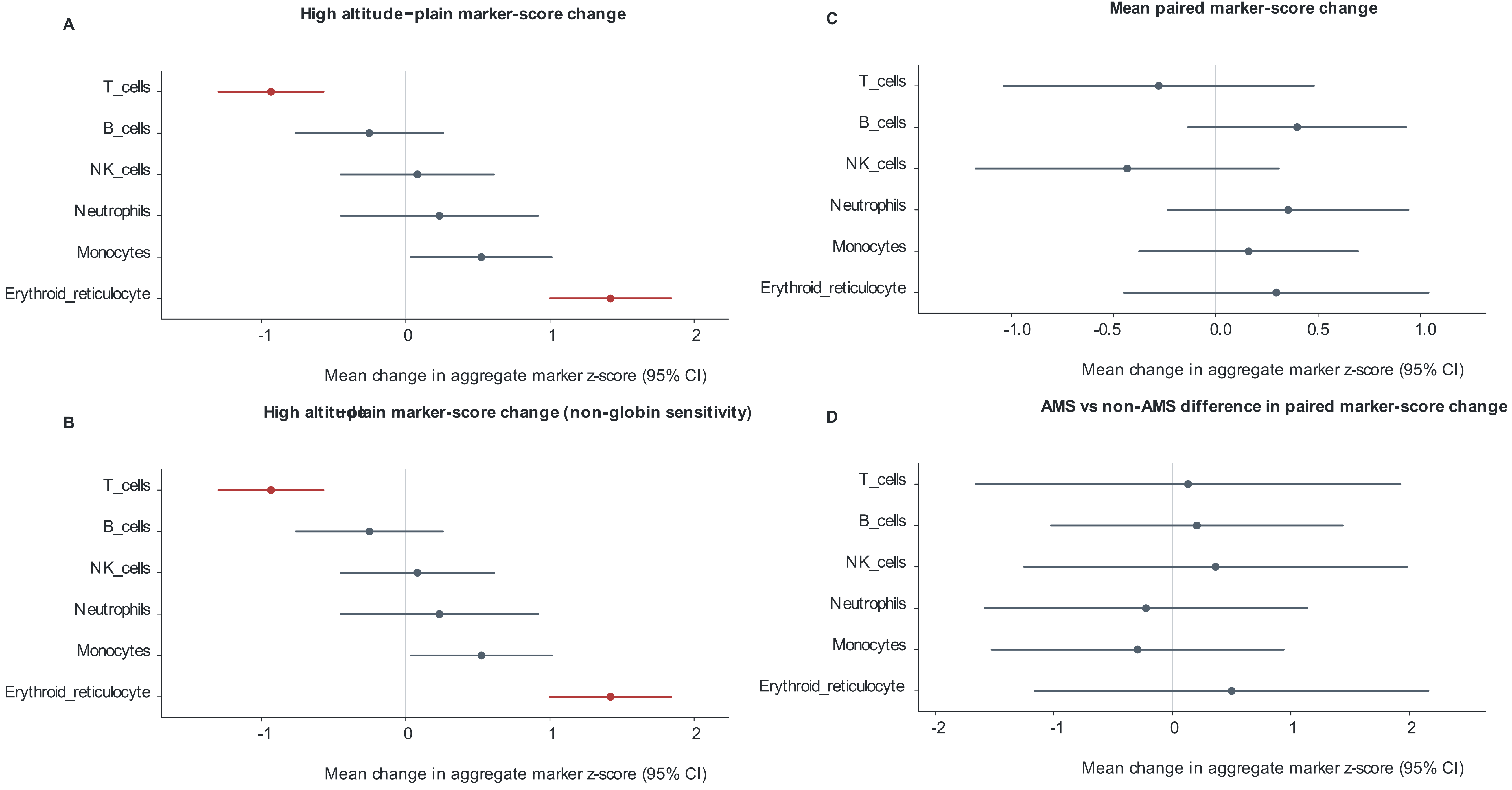

A
High altitude−plain marker-score change
T_cells
B_cells
NK_cells
Neutrophils
Monocytes
Erythroid_reticulocyte
-1
0
1
2
Mean change in aggregate marker z-score (95% CI)
B
High altitude−plain marker-score change (non-globin sensitivity)
T_cells
B_cells
NK_cells
Neutrophils
Monocytes
Erythroid_reticulocyte
-1
0
1
2
Mean change in aggregate marker z-score (95% CI)
C
Mean paired marker-score change
T_cells
B_cells
NK_cells
Neutrophils
Monocytes
Erythroid_reticulocyte
-1.0
-0.5
0.0
0.5
1.0
Mean change in aggregate marker z-score (95% CI)
D
AMS vs non-AMS difference in paired marker-score change
T_cells
B_cells
NK_cells
Neutrophils
Monocytes
Erythroid_reticulocyte
-2
-1
0
1
2
Mean change in aggregate marker z-score (95% CI)
