# Supplementary material for: Integrative Multi-Omics and Network Pharmacology Analyses Reveal Potential Mechanisms of Codonopsis pilosula in Mountain Sickness
Source: Int J Mol Sci. 2026 Oct 18;27(20):9256. doi: 10.3390/ijms27209256 (PMC13705243; doi:10.3390/ijms27209256)
Supplement: Supplementary file 1 [file ijms-27-09256-s001.zip › Supplementary_Figure_S2.pdf]

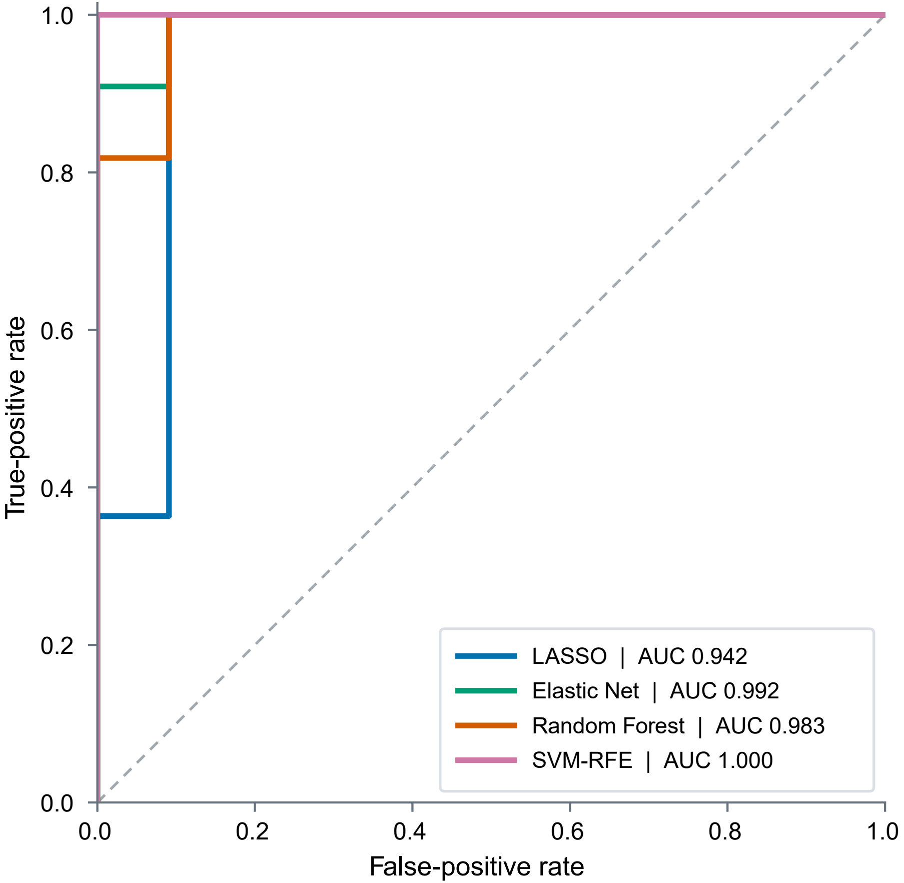
1.0
0.8
0.6
0.4
0.2
0.0
True-positive rate
0.0
0.2
0.4
0.6
0.8
1.0
False-positive rate
LASSO | AUC 0.942
Elastic Net | AUC 0.992
Random Forest | AUC 0.983
SVM-RFE | AUC 1.000
