# Supplementary material for: Integrative Multi-Omics and Network Pharmacology Analyses Reveal Potential Mechanisms of Codonopsis pilosula in Mountain Sickness
Source: Int J Mol Sci. 2026 Oct 18;27(20):9256. doi: 10.3390/ijms27209256 (PMC13705243; doi:10.3390/ijms27209256)
Supplement: Supplementary file 1 [file ijms-27-09256-s001.zip › Supplementary_Figure_S3.pdf]

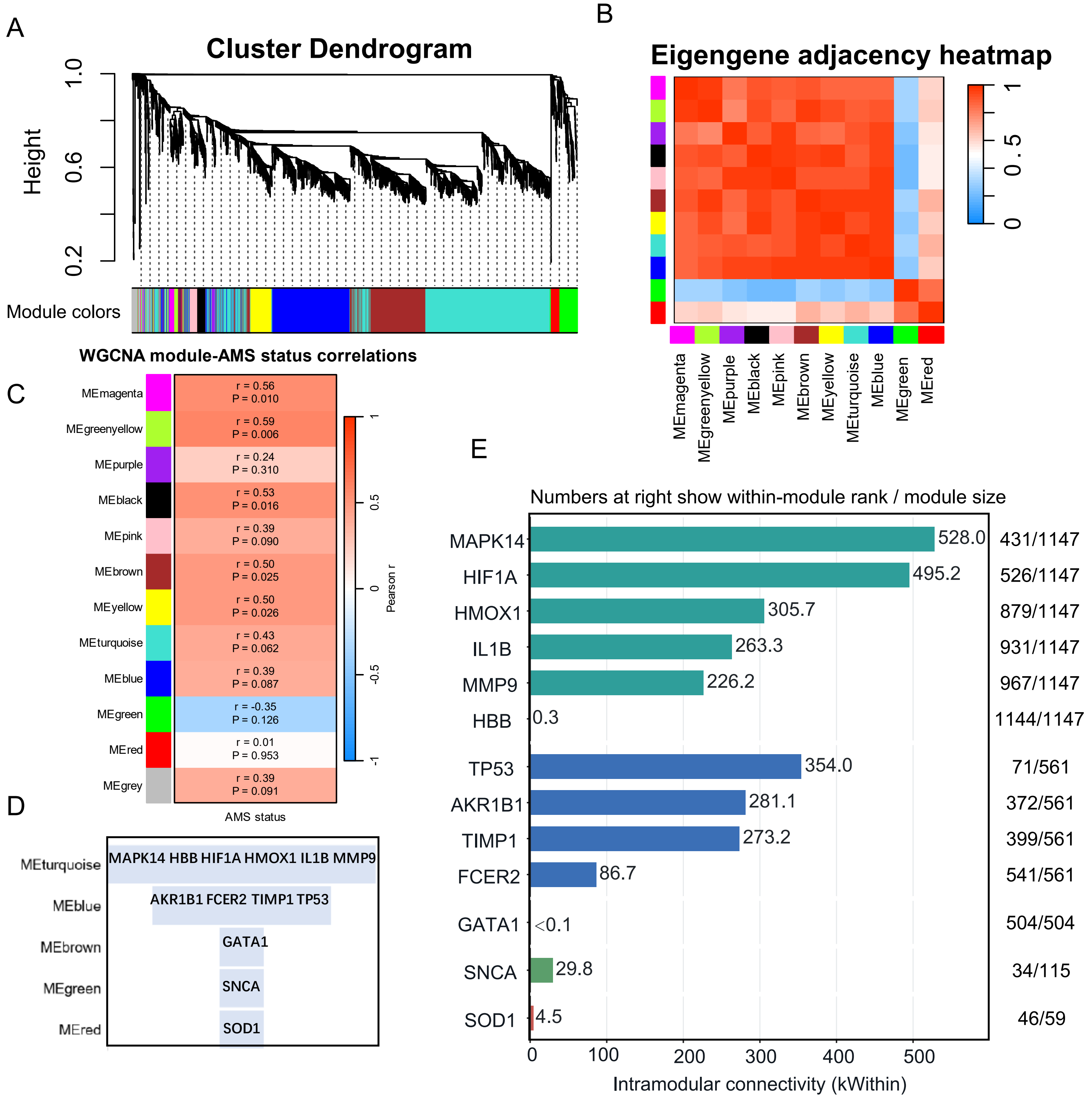

A
Cluster Dendrogram
Height
1.0
0.6
0.2
Module colors
B
Eigengene adjacency heatmap
1
0.5
0
MEmagenta
MEgreenyellow
MEpurple
MEblack
MEpink
MEbrown
MEyellow
MEturquoise
MEblue
MEgreen
MEred
WGCNA module-AMS status correlations
C
MEmagenta r = 0.56 P = 0.010
MEgreenyellow r = 0.59 P = 0.006
MEpurple r = 0.24 P = 0.310
MEblack r = 0.53 P = 0.016
MEpink r = 0.39 P = 0.090
MEbrown r = 0.50 P = 0.025
MEyellow r = 0.50 P = 0.026
MEturquoise r = 0.43 P = 0.062
MEblue r = 0.39 P = 0.087
MEgreen r = -0.35 P = 0.126
MEred r = 0.01 P = 0.953
MEgrey r = 0.39 P = 0.091
AMS status
Pearson r
1
0.5
0
-0.5
-1
D
MEturquoise MAPK14 HBB HIF1A HMOX1 IL1B MMP9
MEblue AKR1B1 FCER2 TIMP1 TP53
MEbrown GATA1
MEgreen SNCA
MEred SOD1
E
Numbers at right show within-module rank / module size
MAPK14 528.0 431/1147
HIF1A 495.2 526/1147
HMOX1 305.7 879/1147
IL1B 263.3 931/1147
MMP9 226.2 967/1147
HBB 0.3 1144/1147
TP53 354.0 71/561
AKR1B1 281.1 372/561
TIMP1 273.2 399/561
FCER2 86.7 541/561
GATA1 <0.1 504/504
SNCA 29.8 34/115
SOD1 4.5 46/59
0
100
200
300
400
500
Intramodular connectivity (kWithin)
